# Primary intraorbital inflammatory lumpy lesion: A rare case report

**DOI:** 10.1097/MD.0000000000037869

**Published:** 2024-04-19

**Authors:** Jinxin Yang, Qianlei Liang, Liang Han, Yan Wang, Yongchuan Guo

**Affiliations:** a Department of Neurosurgery, The Second Hospital of Jilin University, Changchun, China; b Department of Neurosurgery, Chian-Japan Union Hospital of Jilin University, Changchun, China; c Department of Pathology, Chian-Japan Union Hospital of Jilin University, Changchun, China.

**Keywords:** Benign orbital tumor, eosinophilic angiocentric fibrosis, IgG4 negative eosinophilic angiocentric fibrosis, orbital eosinophilic angiocentric fibrosis, orbital lesion

## Abstract

**Rationale:**

Eosinophilic angiocentric fibrosis (EAF) is considered to be a kind of benign IgG4-related disease, and it is more often found in the nasal cavity. We present a pretty rare case of orbital EAF that is unlike any other reported case for this case is an IgG4 negative orbital EAF and successfully treated by the fronto orbitozygomatic approach surgery.

**Patient concerns:**

This is a 68-year-old man from a rural area of Inner Mongolia Autonomous Region, went to our hospital for a 2-month history of vision loss with a local hospital orbital computer tomography which showed that there was a lesion in his left orbit. The inspection of the patient revealed that the patient left eye was protruding outward and the left eyelid unable to complete open or close. And his left eyeball movement had difficulty in all directions. Postoperative pathology diagnosed that this was a case of IgG4-negative EAF case.

**Diagnoses:**

Orbital EAF.

**Interventions:**

Surgical radical resection and postoperative glucocorticoid therapy.

**Outcomes:**

After surgery, the left eye vision of this patient increased to 0.6 tested in the standard logarithmic visual acuity chart. And his left eyeball movement dysfunction and eyeball outward protruding get a partially relief.

**Lessons:**

EAF occurring in the orbit is a very rare disease and immunohistochemical results of EAF can be IgG4 negative.

## 1. Introduction

Eosinophilic Angiocentric Fibrosis (EAF) is considered to be a kind of benign IgG4-related disease diagnosed mainly relying on results of histology.^[1]^ The first known case reported most like EAF was a case of intranasal occurrence granuloma faciale published in 1983 by Douglas K. Holmes et al.^[2]^ In that case, the patient complained of right-sided nasal obstruction, whose right-sided intranasal mass’ gross pathologic report described the tissue as gray-tan. Microscopic examination showed a perivascular infiltrate of eosinophils and histiocytes involving the small arteries and capillaries. And they diagnosed this case as granuloma faciale.^[2]^ Two years after Douglas K. Holmes report, P.F. Roberts^[3]^ reported 3 patients who had developed an obstructive lesion in the upper airways. The 3 patients showed a remarkably similar histological appearance: there were eosinophils cluster around and migrated through the vessel wall, showed degranulation and were accompanied by a variable number of plasma cells and lymphocytes in the peri-vascular space. The more mature foci show early fibrosis characterized by proliferation of spindle-shaped fibroblasts producing a pseudogranulomatous appearance.^[3]^ P.F. Roberts named the lesion as EAF. Besides, P.F. Roberts paper noted that the presence of a striking infiltrate of eosinophils suggests that allergy to an inhaled agent may play a part in the etiology of the disease. And this view is persuasive in the light of papers and current data: there appears to be a higher incidence of allergic and atopic disorders consisting of asthma, drug allergy, environmental allergy, urticaria and allergic rhinitis in patients with EAF than in the general population.^[4]^ And a summary of EAF case reports had been presented showed that the most common anatomic site involved was the nose (77.8%), and the most common manifestation was nasal obstruction (66.7%). Many research even described EAF as a very rare neoplasm of the sinonasal tract and the upper respiratory system.^[5]^ So our case of EAF is very rare for the lesion involving the orbits and the patient of our case complained not for nasal obstruction but for the right eye visual deterioration. This atypical growth site and symptoms can easily lead to misdiagnosis. We have also made some innovations in the treatment of this patient on the common surgical approach, which we hope can be helpful in the treatment of this disease thereafter. These are the rarity of our case and the value of reporting it.

## 2. Patient information

A 68-year-old man from a rural area of Inner Mongolia Autonomous Region presented to their local hospital for a 2-month history of vision loss. And his orbital computer tomography showed that there was a lesion in his left orbit. Then he went to our hospital for further diagnosis and treatment.

This patient has a history of gastric polyps that were surgically treated 2 years ago, but he could not offer more details. The patient had a drinking history of 30 years with an average of 150 grams per day.

## 3. Clinical findings

**Figure 1.**
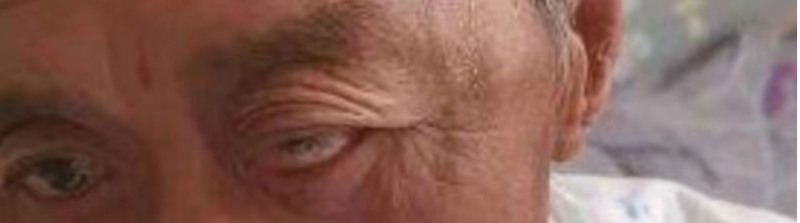
The patient had a protruding left eyeball with severe limitation of motion in all directions.

The physical examination of the patient revealed that the patient left eye was protruding outward with incomplete opening and closing of the eyelid (Fig. 1). And his left eyeball movement had difficulties in all directions, left eye vision was 0.4 tested in the standard logarithmic visual acuity chart.

**Figure 2.**
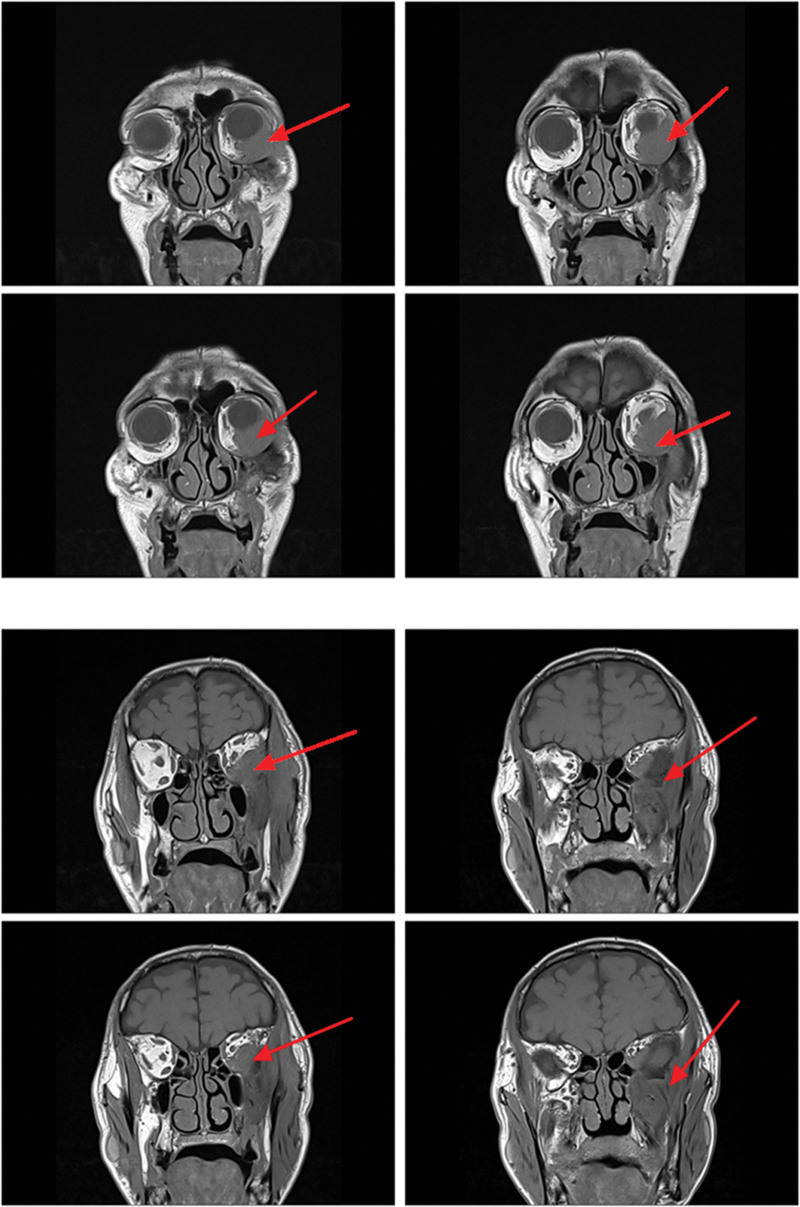
The patient MRI coronal T1 phase left posterior ocular fat space, left lateral ocular, and left inferior ocular muscular space masses are isosignal (red arrows). MRI = magnetic resonance imaging.

**Figure 3.**
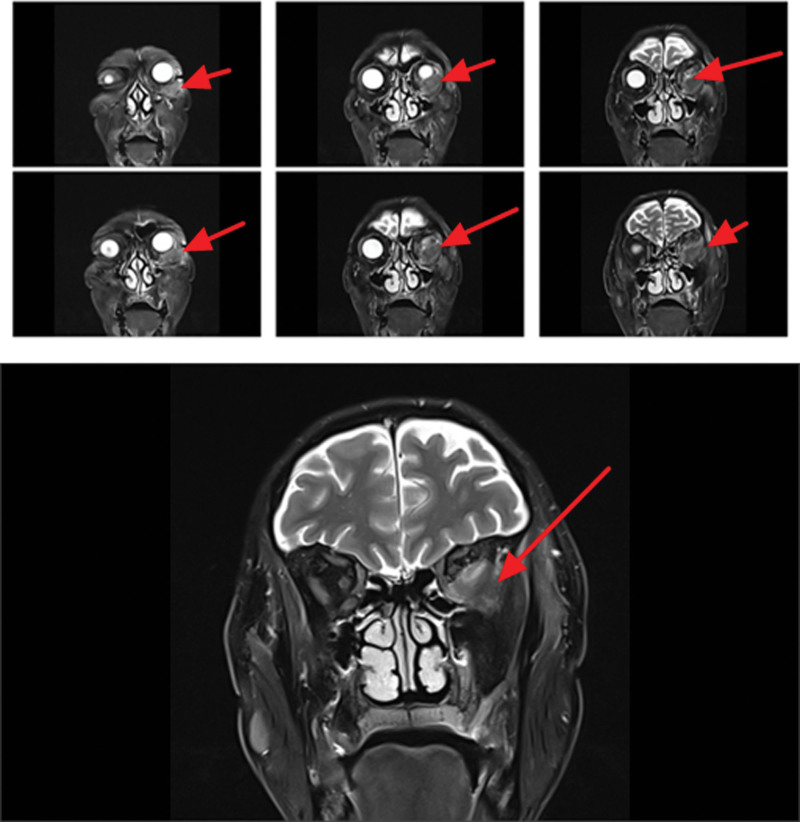
The patient MRI coronal T2 phase left posterior ocular fat space, left lateral ocular, and left infraocular muscular space masses showed slightly high signal (red arrows). MRI = magnetic resonance imaging.

**Figure 4.**
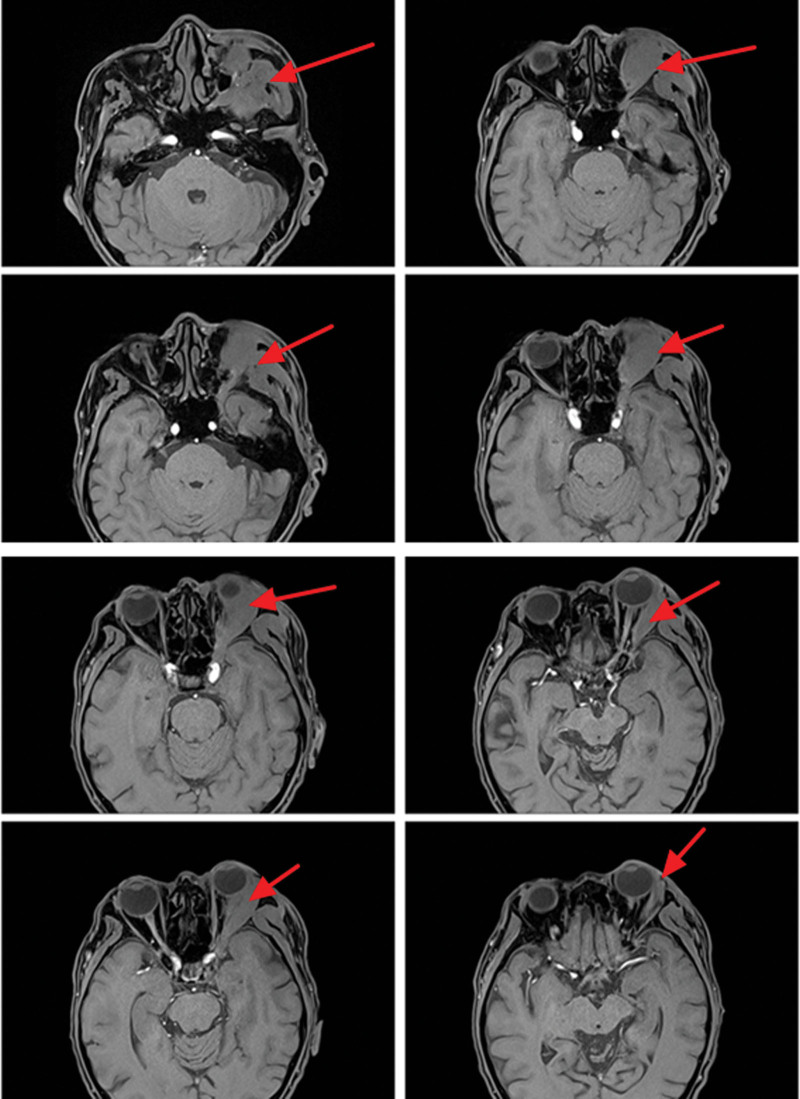
Patient MRI cross-sectional T1 phase with isosignal nonsignificant enhancement of the left posterior fat space, left lateral eye, and left inferior muscle space masses (red arrows). MRI = magnetic resonance imaging.

**Figure 5.**
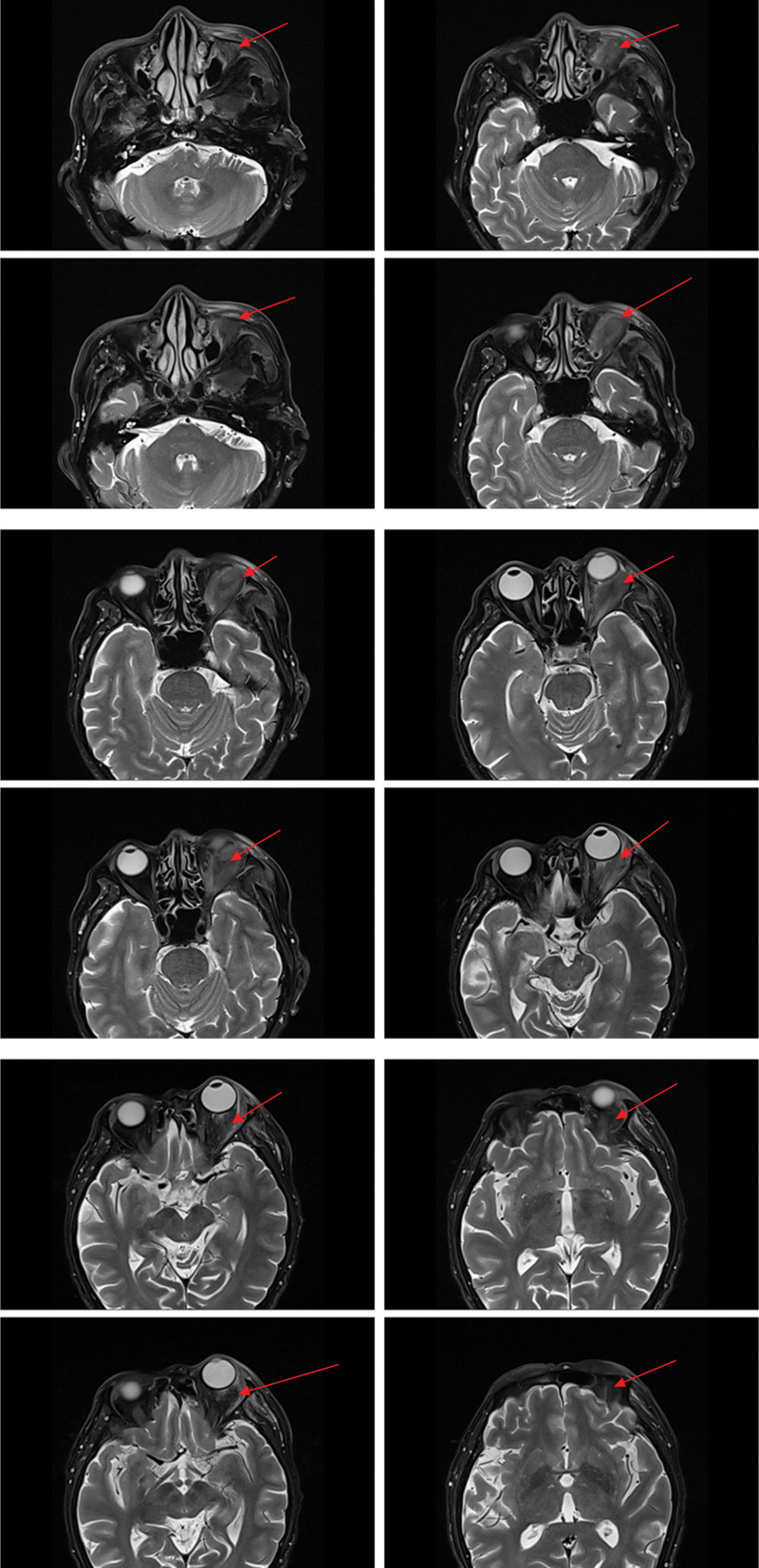
The patient MRI cross-sectional T2 phase showed slightly high signal without significant enhancement of the left posterior fat space, left lateral eyeball, and left inferior muscle space masses (red arrows). MRI = magnetic resonance imaging.

**Figure 6.**
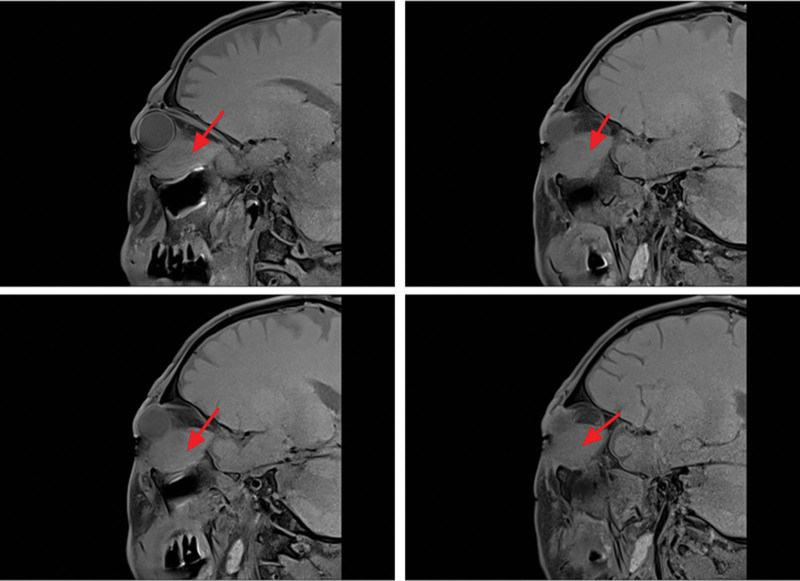
Patient MRI sagittal T1 phase with isosignal (red arrows) in the left posterior fat space, left lateral eye, and left inferior muscle space mass. MRI = magnetic resonance imaging.

**Figure 7.**
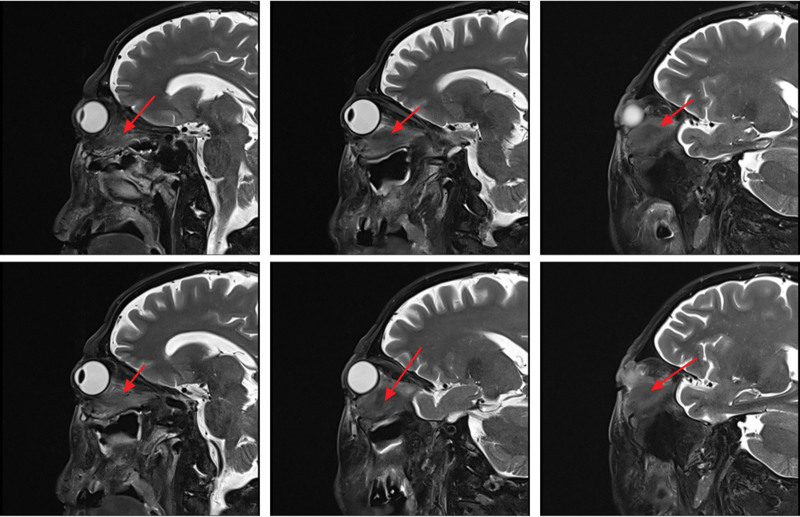
The patient MRI sagittal T2 phase showed slightly high signal (red arrows) in the left posterior fat space, left lateral eyeball, and left inferior muscle space mass. MRI = magnetic resonance imaging.

On magnetic resonance imaging (MRI) plain and enhanced scanning, there was a mass of equal T1 and slightly long T2 signals in the fat space behind the left eyeball, the lateral side of the left eyeball, and the muscle space below the left eyeball. And there was obvious homogeneous enhancement on enhancement scanning, and the size of enhancement signals was about 5.1*2.8*2.5 cm, and the lesion cannot be clearly demarcated from the optic nerve, posterior globe of the left eyeball, and the posterolateral wall of the left eyeball. The left external and inferior rectus muscles and lacrimal gland could not be clearly identified on the MRI because of the invasion of the tumor. Part of the tumor broke through the lateral orbital wall outwardly to the left temporal fossa, invaded the left temporal muscle, and grew toward the orbital apex, invaded the left cavernous sinus, and the left optic chiasm could be seen to be widened on the nuclear magnetic image, and the lesion protruded backward and downward into the pterygopalatine fossa through the left infraorbital fissure, with a slight thickening and enhancement of the meninges in front of the temporal pole. The meninges are slightly thickened and are intensified during the enhancement phase (Figs. 2–7).

## 4. Diagnostic assessment

**Figure 8.**
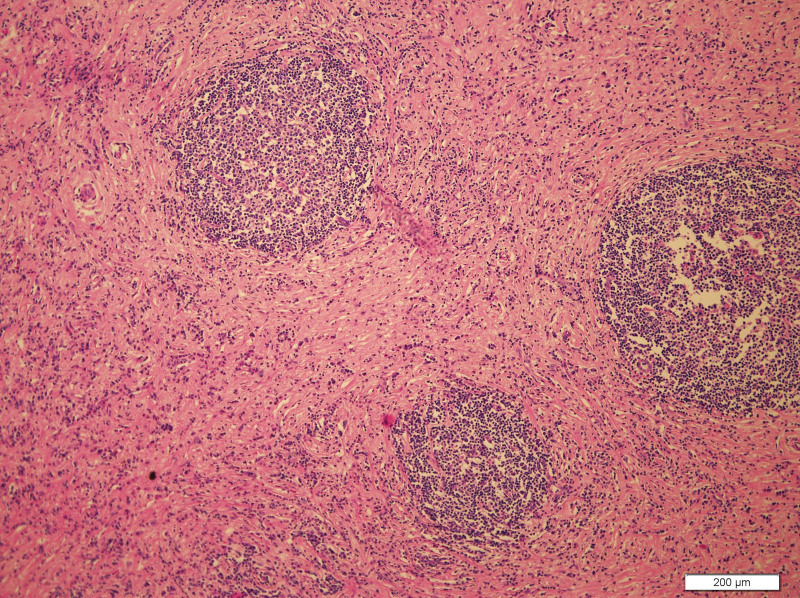
HE staining of patient mass (200×). HE staining = hematoxylin and eosin staining.

**Figure 9.**
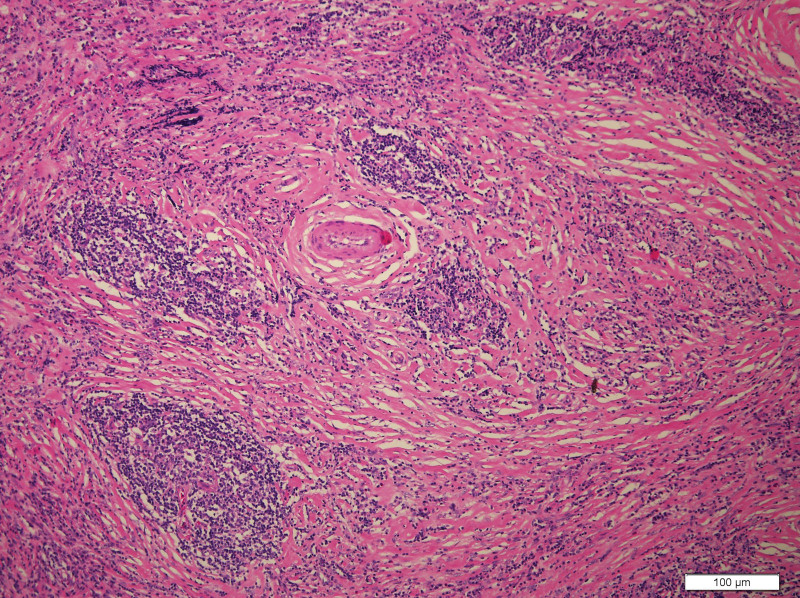
HE staining of patient mass (100×). HE staining = hematoxylin and eosin staining.

**Figure 10.**
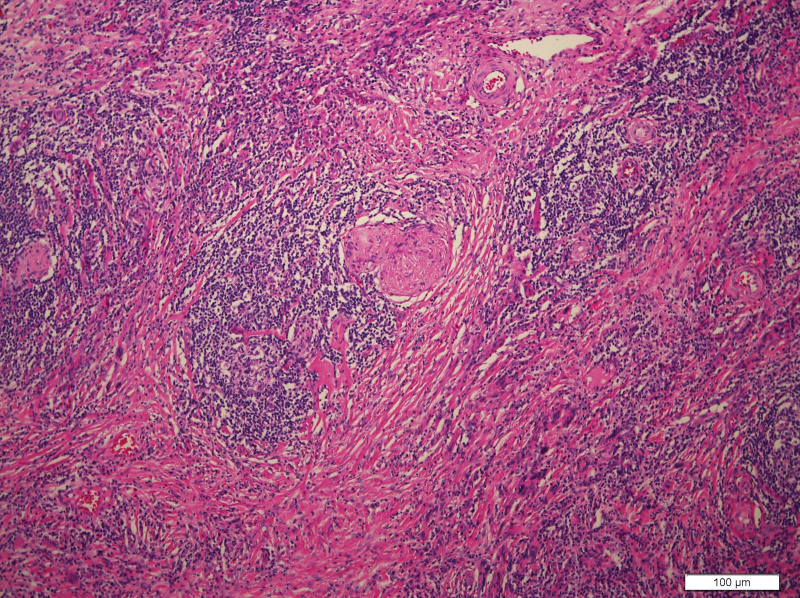
HE staining of patient mass (100×). HE staining = hematoxylin and eosin staining.

**Figure 11.**
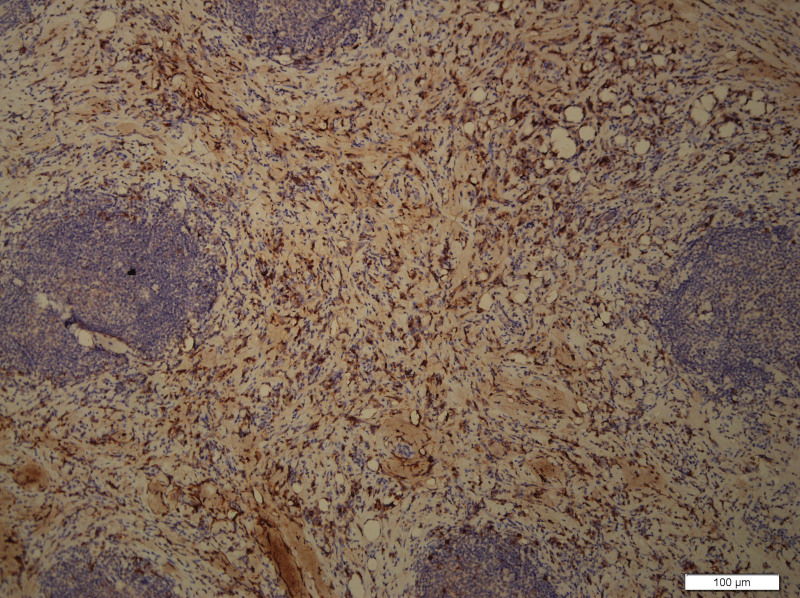
Immunohistochemistry is positive of EMA. EMA = epithelial cell adhesion molecule).

**Figure 12.**
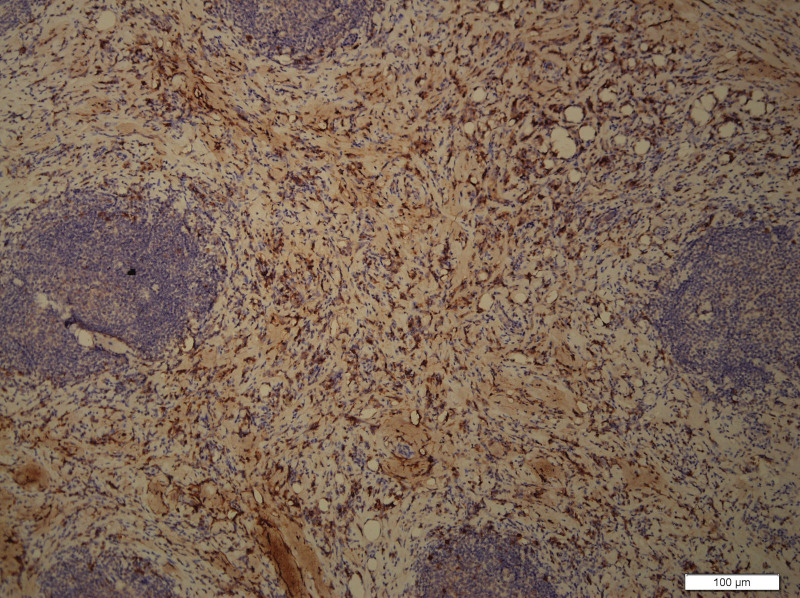
Immunohistochemistry showed positive CD138.

**Figure 13.**
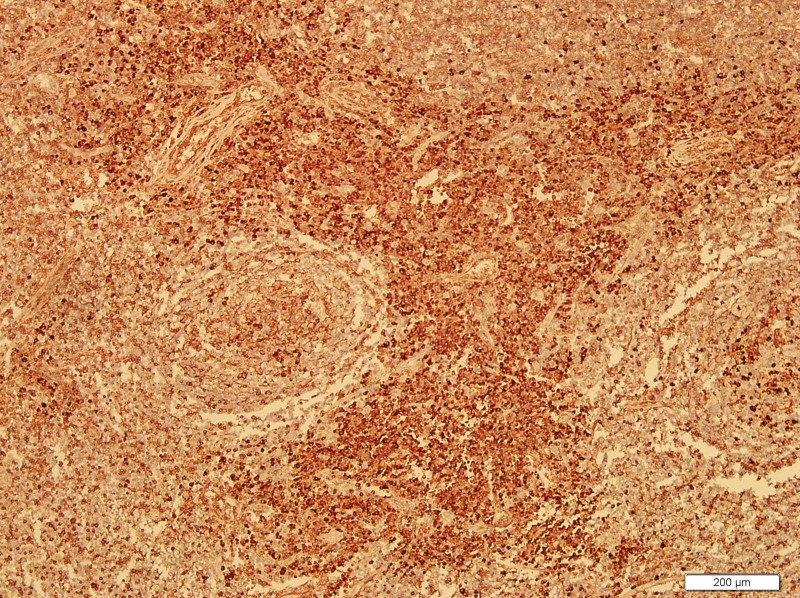
Immunohistochemistry showed positive κ.

**Figure 14.**
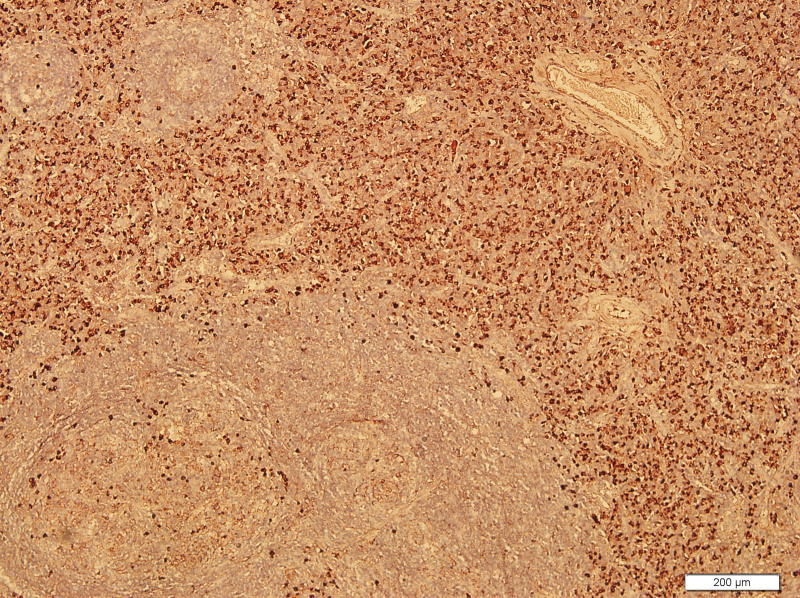
Immunohistochemistry showed λ-positive.

**Figure 15.**
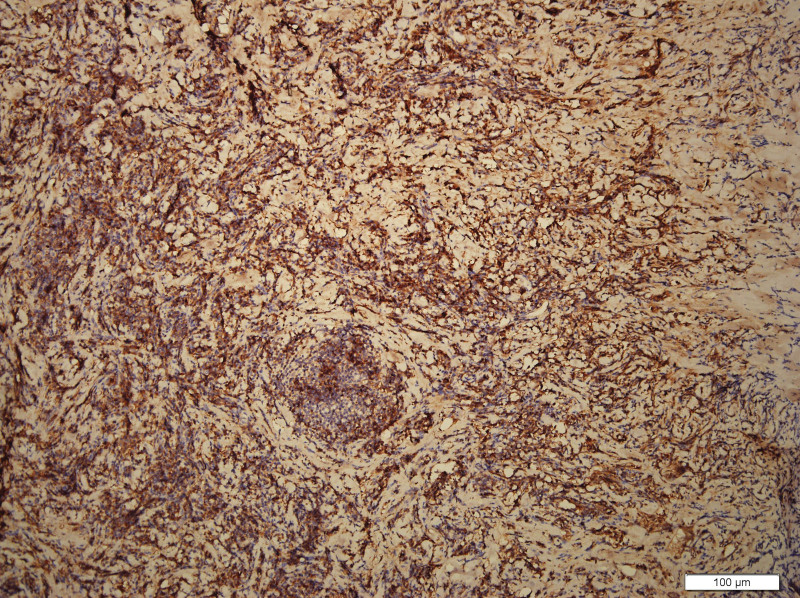
Immunohistochemistry showed positive IgG.

**Figure 16.**
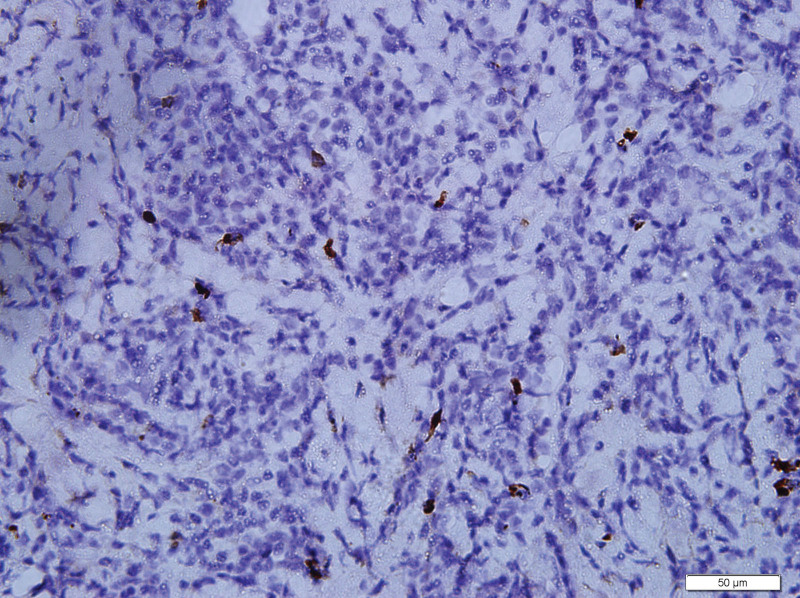
Immunohistochemistry showed negative IgG4.

What eventually established the diagnosis is the result of pathology. The histology demonstrated the blood vessels surrounded by dense fibrosis thickening giving an onion skin-like appearance, with plasma cells, eosinophils and lymphoid follicles’ infiltration(Figs. 8–10). Immunohistochemistry shows: EMA positive (Fig. 11), CD138positive (Fig. 12), κ positive (Fig. 13), λ positive (Fig. 14), IgG positive (Fig. 15), and IgG4 negative (Fig.16).

## 5. Therapeutic intervention

Since there were no contraindications, we performed surgery under general anesthesia for him after preoperative preparation.

**Figure 17.**
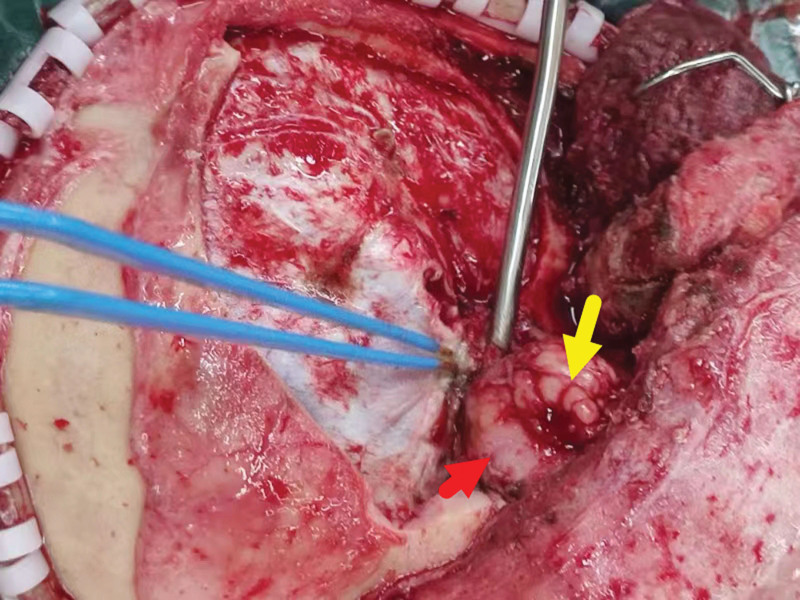
This is an intraoperative photo, shows the tumor (yellow arrow) and the left side of the eye ball (red arrow).

**Figure 18.**
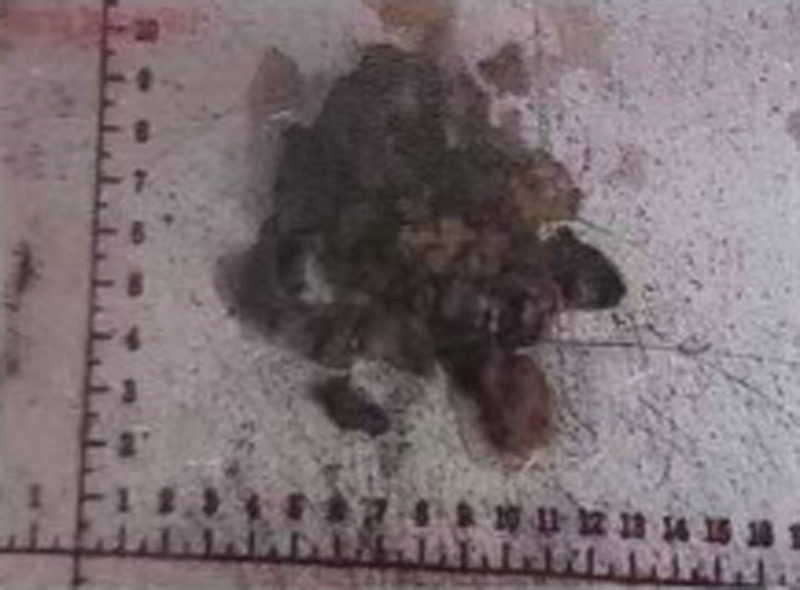
Tumor tissue removed in chunks.

What we want to introduce is our surgery methods. We used the fronto orbitozygomatic approach to remove the superior orbital wall and the zygomatic arch, found that the entire temporal fossa and the muscles in the infratemporal fossa had been invaded by the lesion and were present as hard-textured (Fig. 17). The tumor occupied the space below and temporal side of the eye, causing the eye ball shifted to the superior and nasal side of the orbit. The tumor also protruding into the infratemporal fossa and invading the bone of the orbital wall. Besides, we noticed that the texture of the tumor was tough with a poor blood supply (Fig. 17). We carefully separated the tumor from normal tissue and resected the tumor in piecemeal resection and the tumor was eventually removed completely (Fig. 18). Postoperative treatment with methylprednisolone.

## 6. Follow-up and outcomes

**Figure 19.**
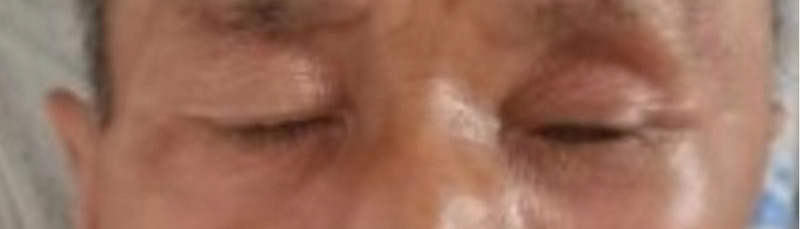
Postoperatively, the patient left ocular motility dysfunction and outward protrusion of the eyeball were partially relieved.

**Figure 20.**
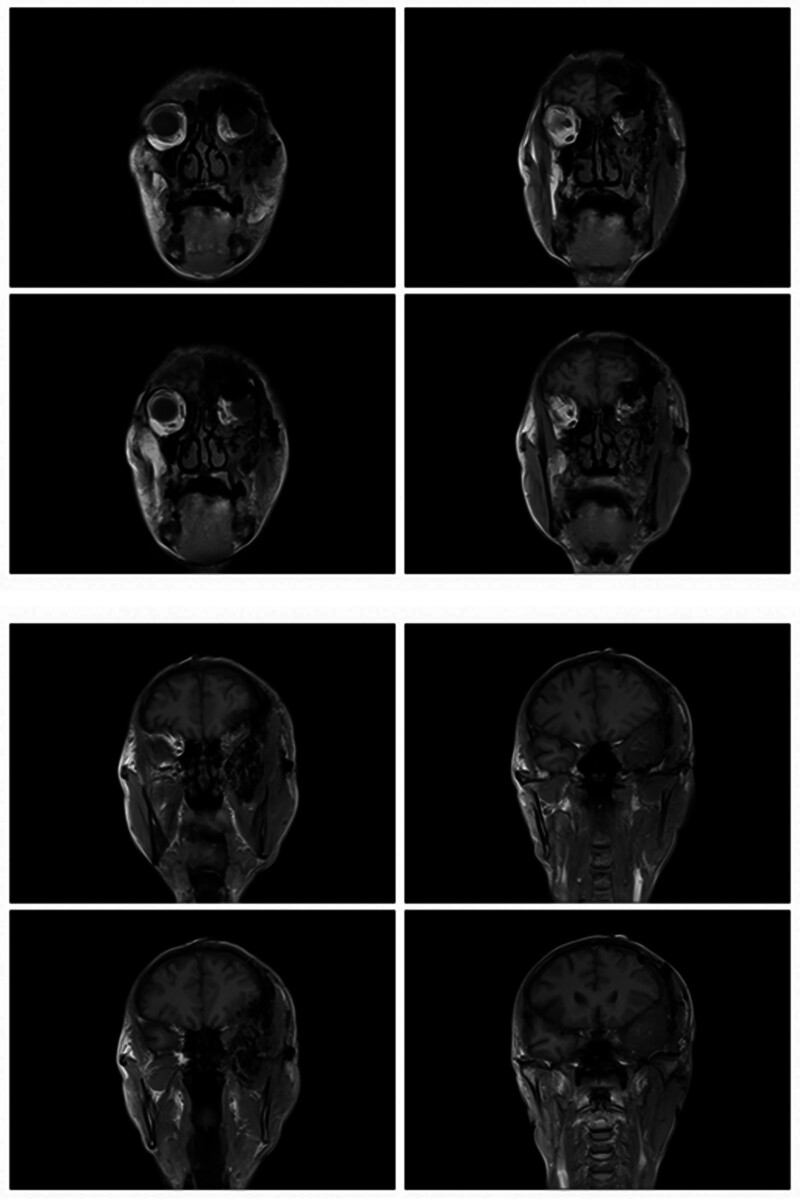
Patient postoperative MRI coronal T1 phase. MRI = magnetic resonance imaging.

**Figure 21.**
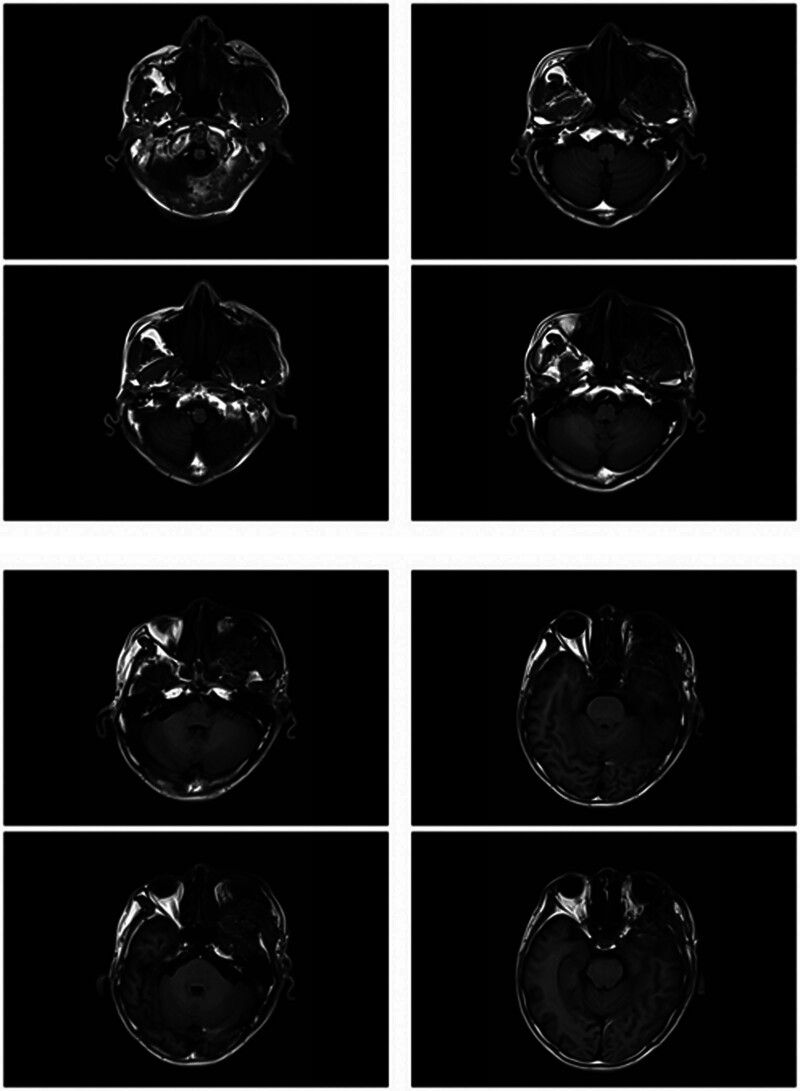
Patient postoperative MRI cross-sectional T1 phase. MRI = magnetic resonance imaging.

**Figure 22.**
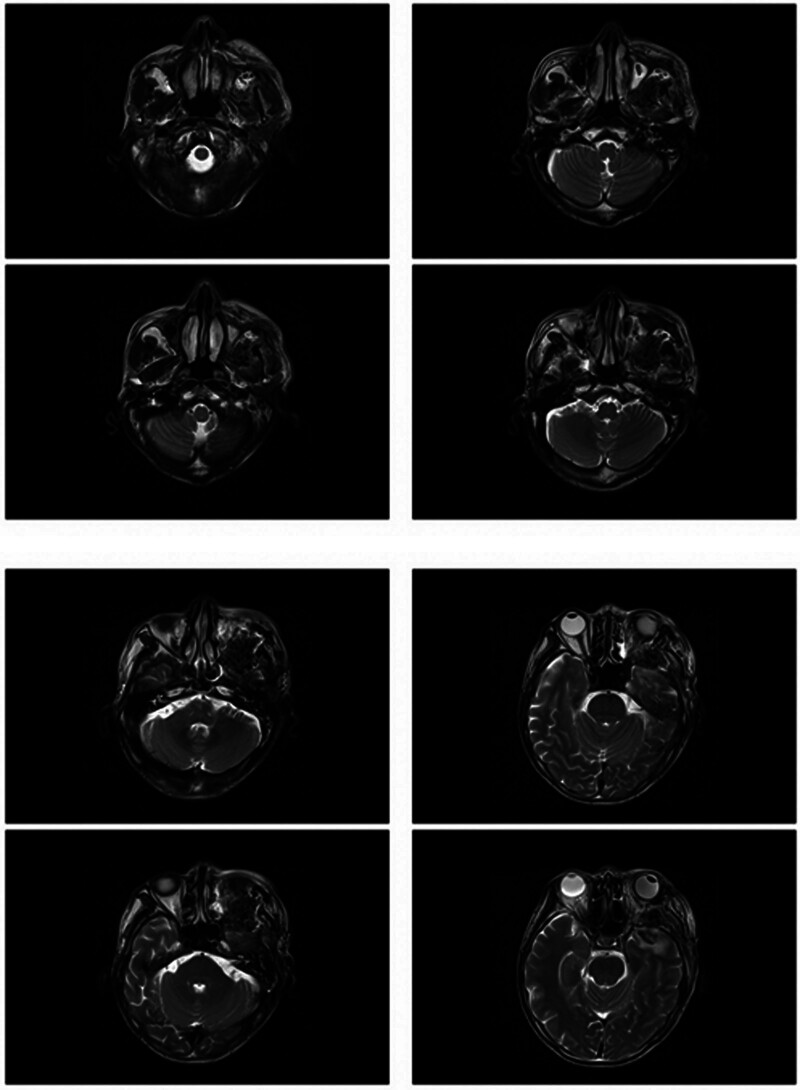
Patient postoperative MRI cross-sectional T2 phase. MRI = magnetic resonance imaging.

**Figure 23.**
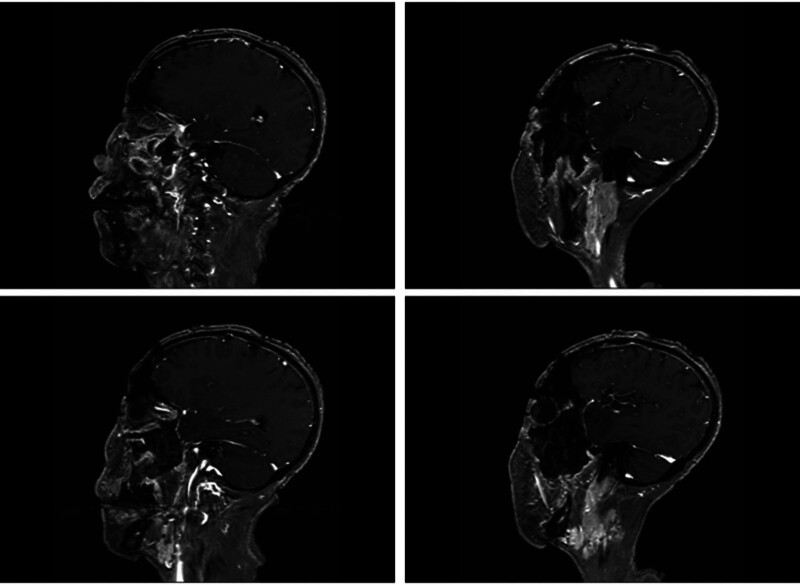
Patient postoperative MRI-enhanced scanning cross-sectional T1 phase. MRI = magnetic resonance imaging.

After surgery, the left eye vision of this patient was 0.6 tested in the standard logarithmic visual acuity chart. His left eyeball abduction movements gained a significant improvement, but his left eyeball movement in all directions still worse than normal people and eyeball outward protruding get partially relief (still being a little protruding) (Fig. 19). Here are MRI plain and enhanced scanning after the surgery 5 days (Figs. 20–23). And postoperative pathology revealed that it is a rare case of IgG4-negative orbital EAF. We followed up him for 2 years after he discharged from our hospital, and there were no signs of the tumors’ recurrence (the patient did not have complaints that deterioration of vision or recurrence of eyeball obvious protruding, but he also did not want to go to hospital to do more times of review for economic reason.

## 7. Discussion

Orbital EAF various clinical manifestations include diffuse inflammatory lesions or localized mass lesions, and it is also invasive, spreading easily to surrounding tissues and often causing bone damage. Besides, the symptoms that EAF exhibits are strongly tied to the anatomical site, which is mostly the nose, sinuses, and subglottic region. As a result, the primary symptoms were growing nasal blockage and stuffiness, breathing difficulty, epistaxis, and discomfort. These vague symptoms are the primary cause of the normally protracted delay in diagnosis of this disease.^[6,7]^ So the signs and symptoms of Orbital EAF are also probably more related to compression. And here we give some examples from the literature reported by other scholars: Igal Leibovitch case report presented the patient with a 6-week history of right periorbital edema and painless proptosis, with a 3-mm right nonaxial proptosis and 2 mm lateral globe displacement was noted.^[8]^ One another article put out that proptosis is less likely to happen in the orbital EAF patient,^[9]^ but eyeball proptosis in our case was significant. We have summarized the signs and symptoms of reported orbital EAF in Table 1.

**Table 1:** Summarizing EAF cases of orbital origination or orbital involvement described in the literature.

Authors	Yr	Sex, age	Site	Signs and symptoms	Treatment	Follow-up time and result
Igal Leibovitch et al	2006	61,male	Right orbit	Right periorbital edema and painless proptosis	Surgery, prednisolone,	6 mo:no significant change in his clinical examination
Hayyam Kiratli et al	2008	30,female	Right orbit	Right ipsilateral epiphora, diplopia on left gaze and adduction of the right eye was slightly restricted, displacement of the right eye	Surgery, fluocortolon and oral desloratadine	
Muhammad Azam et al	2010	35,female	Beneath the right lower eyelid outside the orbit	Pain and right lower eyelid swelling	Surgery	8 mo with asymptomatic
Apostolos Karligkiotis et al	2014	46,male	The right middle meatus and the right orbit	Lateral globe displacement, right unilateral nasal obstruction, and supraorbital headache	surgery	
Yasuhiro Takahashi et al	2014	43,male	The right frontal, maxillary, ethmoidal, sphenoid sinuses and orbit invaded	Nasal obstruction, right visual loss and eyelid swelling, Extraocular muscle motility	Surgery, methylprednisolone, diaminodiphenyl sulfone, cyclophosphamide	1.5 yr with no evidence of recurrence
Mohammad Faramarzi et al	2015	35,male	Left maxillary sinus	Slowly progressive orbital swelling	Surgery	1 yr with no evidence of recurrence
Santhi Radhakrishnan et al	2015	38,female	In the inferolateral aspect of the right orbit extending from the rim to just behind the globe	Right inferior orbital painless mass with limited abduction in the right eye	Surgical debulking of the mass and postoperatively oral prednisolone of orbital disease	Last follow-up time not given, completely free of signs and symptoms
Adam P. Lloyd	2015	45,male	The left orbital apex, encompassing the intracranial optic nerve and involving the left cavernous sinus and pterygopalatine fossa	Progressive visual loss in his left eye, retroorbital pain	Surgery	Follow-up time and result not mentioned
S. Gorostis et al	2017	61,male	Right orbit	Headache, nasal obstruction and symptoms in the right eye: retroorbital pain, watering, exophthalmos, palpebral edema, progressive deterioration of visual acuity resulting in blindness	Surgery, corticosteroid and rituximab	Twelve-mo follow-up with complete resolution of pain, no further episodes of exophthalmos and discontinuation of corticosteroid therapy
Nicole Legare et al	2018	58,male	A solid mass along the nasal soft tissue extending bilaterally into the medial periorbital soft tissue to the level of the lacrimal sac	Left eyelid swelling, bloody nasal discharge	Prednisone and rituximab	Follow-up 6 mo: the periocular mass, edema and bloody nasal discharge get a sustained relief
Antonio Ramos Suárez et al	2018	43,male	Both the lateral areas of the orbit	Headache and horizontal diplopia, mydriasis, eye movement dysfunction	Steroids, cyclophosphamide, methotrexate Steroids, cyclophosphamide, methotrexate	Follow-up 2 mo: ocular motility almost returned to normal, the anterior pole of the right eye was normal and chemosis and proptosis in the left had significantly improved, the left eye showed no improvement; the Marcus Gunn pupil and anisocoria persisted in the left eye
Helen-Melissa Heedari et al	2021	69,male	Left orbital	Left orbital periorbital edema, epiphora and retroocular pain	Methylprednisolone	4 mo: periorbital edema decreased, without improvement of the ocular motility
Rui Liu et al	2023	55,female	Right outer orbit	Recurrent swelling of the right lower eyelid	Surgery	Not mentioned

EAF = eosinophilic angiocentric fibrosis.

The imaging of EAF also is nonspecific and untypically either in computer tomography or MRI, and usually it shows a well-circumscribed submucosal soft tissue density mass.^[6]^ For example, soft tissue thickening of the septum and lateral nasal walls, as well as clouding or opacification of the sinuses, are common imaging findings in EAF.^[6]^ Igal Leibovitch, 1 case report author of Orbital EAF, described their case MRI showed the mass was mainly orbital, with no obvious nasal involvement.^[8]^ Another case of orbital EAF case MRI, a contrast-enhanced coronal scan shows a thick rind of mildly enhancing soft tissue around right eyeball and soft tissue thickening seen isointense to the muscle, around right eyeball.^[10]^ Magnetic imaging of our EAF case showed isosignal with the muscle, and was significantly enhanced by contrast.

The characteristic histologic feature of EAF are as follows: a dense fibrotic stroma with a perivascular “onion skin-like” whorling pattern and a dense inflammatory infiltrate that the major cells are eosinophils, consisting of lymphocytes, plasma cells, and some neutrophils.^[11]^ Microscopic pathology in our case showed proliferative fibrous tissue, eosinophils, lymphocytes and plasma cells. And they distributed in concentric circles around the vessel. On the basis of these typical histological characteristics, we were able to diagnose the patient as an orbital EAF.

However, our case IgG4 showed in immunohistochemical result is negative. But the definition of EAF usually descripted as a kind of rare, benign IgG4-related disease (IgG4-RD) diagnosed by histology,^[1]^ which require that IgG4+/IgG plasma cell ratio >40% and more than 10 IgG4 + plasma cells per high-power field.^[1]^ There are no pathologists who have studied and explained why some patients are negative for IgG4, which is an interesting and worthwhile question. We also summarized the immunohistochemical results of reported orbital EAF in Table 2.

**Table 2:** Summarizing EAF cases’ immunohistochemical results of orbital origination or orbital involvement described in the literature.

Authors	Immunohistochemical results
Igal Leibovitch et al	No immunohistochemical results
Hayyam Kiratli et al	No immunohistochemical results
Muhammad Azam et al	No immunohistochemical results
Apostolos Karligkiotis et al	No immunohistochemical results
Yasuhiro Takahashi et al	Few IgG4-positive plasma cells
Mohammad Faramarzi et al	No immunohistochemical results
Santhi Radhakrishnan et al	No immunohistochemical results
Adam P. Lloyd	A mixed inflammatory cell population includes scattered mature plasma cells, a proportion of which show avid cytoplasmic IgG4 immunopositivity. There is some background reactivity, possibly representing serum IgG4
S. Gorostis et al	Plasma cells presented positive IgG4 labeling and an IgG4/IgG ratio >40%. Serum IgG4 levels were higher than 1.35 g/L (1.910)
Nicole Legare et al	The IgGr:IgG was 1:1 and the cells were not immunoreactive for S100 protein, keratin or smooth muscle actin
Antonio Ramos Suárez et al	No IgG deposits were visible on immunofluoroscopy
Helen-Melissa Heedari et al	IgG4 staining was mainly positive and IgG4 plasma cells were about 40% of all the inflammatory cells
Rui Liu et al	Negative for immunoglobulin G4 (IgG4) and positive for IgG, CD34, κ, and λ

EAF = eosinophilic angiocentric fibrosis.

Differential diagnoses including granuloma faciale, Wegener granulomatosis, Churg-Strauss syndrome, and Kimura disease are discussed by many other papers,^[12]^ so we would not discuss EAF differential diagnoses in this article.

Surgical resection could be the first choice of treatment, while there are recurrent cases of EAF after surgery,^[13–16]^ and a first-time incomplete surgical resection or even a biopsy may contribute to the development of the tumor in a negative way.^[17]^ Besides, there are scholars view that systemic and topical corticosteroids as ineffective.^[18]^ But in any way, it is hard to diagnosis EAF without pathological results, and glucocorticoids appear to be effective initially.^[19]^ Therefore, combined surgery to remove as much of the lesion as possible with glucocorticoid or other medicines like azathioprine, mycophenolate mofetil, and methotrexate as remission-maintenance drugs may be the best option at present.

We summarize the surgical experience of this patient: firstly, adequate exposure of the lesion according to preoperative MRI can ensure maximal tumor resection. Using the fronto orbitozygomatic approach to open the cranium, and then use the grinding drill to grind all the bones of the middle skull base and the lateral wall of the cavernous sinus, at the same time, cut the falciform ligament, and cut the dura at the proximal fissure for about half a centimeter, and then adjust the focus to the carotid artery pool along this area using a microscope, releasing the cerebrospinal fluid to make the brain tissues to collapse, so that the tumor can be exposed sufficiently at the level of the middle skull base, and then resected the tumor according to a certain sequence. The sequence was medio-lateral orbital, infraorbital wall, and after removing the intraorbital tumor then turning to the infratemporal fossa. Secondly, according to the texture of tumor to choose cavitron ultrasonic surgical aspirator, monopolar knives scissors and sharp knives. Thirdly localized bone with abnormal hyperplasia is considered to be invasive and can be removed by grinding drill. Fourthly, the anatomical structure should be strictly mastered, especially the neurovascular which has been mutated and displaced. We hope that our experience will inform our peers in the surgical removal of similar tumors.

On the end of our article, we also want to point out the limitations of this research. First, this case lacks laboratory tests for serum IgG4. Based on published literature, the diagnosis of IgG4-RD relies on the basis of clinical, radiological (localized or diffuse hypertrophy of one or more organs classically involved in the course of IgG4-RD), laboratory (serum IgG4 greater than or equal to 135 mg/dL) and immunohistochemical criteria (intense polyclonal round cell infiltration and fibrosis, IgG4 + plasma cell infiltration: IgG4+/IgG plasma cell ratio >40% and more than 10 IgG4 + plasma cells per high-power field).^[1]^ Since this patient was not sure that what kind of disease was preoperatively, taking a serum IgG4 test was not thought of before operation. We hope that our colleagues who read this article will learn from our lesson that in patients with orbital occupations of unknown cause, to perform a serum IgG4 test could be helpful for clues to diagnose the disease. Secondly, although the article mentions a 2-year follow-up showing no signs of recurrence, more detailed follow-up data, such as long-term changes in patients’ vision recovery, are lacking. Because follow-up is constrained by various factors including the patient financial reasons, and as far as the follow-up results of this patient are concerned, the treatment for this patient (especially the operation method) achieved a satisfactory therapeutic outcome. Besides, we look forward to the academic community having operation method comparisons for this kind of type orbital disease in the future.

## Author contributions

**Conceptualization:** Jinxin Yang.

**Formal analysis:** Jinxin Yang.

**Funding acquisition:** Yongchuan Guo.

**Investigation:** Jinxin Yang.

**Methodology:** Jinxin Yang.

**Project administration:** Jinxin Yang.

**Resources:** Jinxin Yang, Qianlei Liang, Wang Yan, Han Liang.

**Software:** Jinxin Yang.

**Supervision:** Yongchuan Guo.

**Writing – original draft:** Jinxin Yang.

**Writing – review & editing:** Jinxin Yang, Qianlei Liang.

## References

[1] Gorostis S Bacha M Gravier S. Right ethmoid eosinophilic angiocentric fibrosis with orbital extension. Eur Ann Otorhinolaryngol Head Neck Dis. 2017;134:351–4. 28359733 10.1016/j.anorl.2017.02.012

[2] Holmes DK Panje WR. Intranasal granuloma faciale. Am J Otolaryngol. 1983;4:184–6. 6881462 10.1016/s0196-0709(83)80041-6

[3] Roberts PF McCann BG. Eosinophilic angiocentric fibrosis of the upper respiratory tract: a mucosal variant of granuloma faciale? A report of three cases. Histopathology. 1985;9:1217–25. 4085985 10.1111/j.1365-2559.1985.tb02801.x

[4] Magro CM Dyrsen M. Angiocentric lesions of the head and neck. Head Neck Pathol. 2008;2:116–30. 20614334 10.1007/s12105-008-0049-2 PMC2807549

[5] Onder S Sungur A. Eosinophilic angiocentric fibrosis: an unusual entity of the sinonasal tract. Arch Pathol Lab Med. 2004;128:90–1. 14692804 10.5858/2004-128-90-EAF

[6] Thompson LD Heffner DK. Sinonasal tract eosinophilic angiocentric fibrosis. A report of three cases. Am J Clin Pathol. 2001;115:243–8. 11211613 10.1309/7D97-83KY-6NW2-5608

[7] Owa AO Boyle S Gallimore AP. Eosinophilic angiocentric fibrosis as a cause of nasal obstruction. Rhinology. 2002;40:41–3. 12012953

[8] Leibovitch I James CL Wormald PJ. Orbital eosinophilic angiocentric fibrosis case report and review of the literature. Ophthalmology. 2006;113:148–52. 16324746 10.1016/j.ophtha.2005.09.035

[9] Jain R Robblee JV O’Sullivan-Mejia E. Sinonasal eosinophilic angiocentric fibrosis: a report of four cases and review of literature. Head Neck Pathol. 2008;2:309–15. 20614301 10.1007/s12105-008-0077-y PMC2807588

[10] Azam M Husen YA Hasan SH. Eosinophilic angiocentric fibrosis of orbit. Indian J Pathol Microbiol. 2010;53:850–2. 21045443 10.4103/0377-4929.72086

[11] Javadirad E Roozbahani NE Sadafi S. Eosinophilic angiocentric fibrosis of the sinonasal tract: a case report and review of the literature. J Int Med Res. 2022;50:3000605221126039. 36172997 10.1177/03000605221126039 PMC9528026

[12] Sazgar AA Kia S Akbari A. Nasal framework reconstruction in patient with eosinophilic angiocentric fibrosis. Indian J Otolaryngol Head Neck Surg. 2019;71:2031–5. 31763288 10.1007/s12070-018-1453-x PMC6848623

[13] Paun S Lund VJ Gallimore A. Nasal fibrosis: long-term follow up of four cases of eosinophilic angiocentric fibrosis. J Laryngol Otol. 2005;119:119–24. 15829064 10.1258/0022215053419989

[14] Slovik Y Putterman M Nash M. Eosinophilic angiocentric fibrosis of the sinonasal tract in a male patient with chronic bowel inflammation. Am J Rhinol. 2006;20:91–4. 16539302

[15] Nguyen DB Alex JC Calhoun B. Eosinophilic angiocentric fibrosis in a patient with nasal obstruction. Ear Nose Throat J. 2004;83:183–6. 15086013

[16] Narayan J Douglas-Jones AG. Eosinophilic angiocentric fibrosis and granuloma faciale: analysis of cellular infiltrate and review of literature. Ann Otol Rhinol Laryngol. 2005;114(1 Pt 1):35–42. 15697160 10.1177/000348940511400107

[17] Li Y Liu H Han D. Eosinophilic angiocentric fibrosis of the nasal septum. Case Rep Otolaryngol. 2013;2013:267285. 23634315 10.1155/2013/267285 PMC3619668

[18] Faramarzi M Dadgarnia MH Moghimi M. Nasal eosinophilic angiocentric fibrosis with orbital extension. Head Neck Pathol. 2015;9:426–9. 25601283 10.1007/s12105-014-0605-x PMC4542801

[19] Stone JH Zen Y Deshpande V. IgG4-related disease. N Engl J Med. 2012;366:539–51. 22316447 10.1056/NEJMra1104650

